# Corrigendum to Global research status of localised scleroderma reported over the period 1993–2022: A 30‐year bibliometric analysis

**DOI:** 10.1111/iwj.14868

**Published:** 2024-04-25

Li ZM, Li TH, Li ZJ, Wang LQ, Long X, Huang JZ. Global research status of localized scleroderma reported over the period 1993–2022: a 30‐year bibliometric analysis. *Int Wound J*. 2024;21(1):e14559. doi: 10.1111/iwj.14559

In the funding information section, the text currently reads “This work was funded by the Medical Science and Health Technology Innovation Project (2022‐I2M‐1‐003).” It should have been “This work was funded by the National Key R&D Program of China (2020YFE0201600) and the National High Level Hospital Clinical Research Funding (2022‐PUMCH‐C‐025, 2022‐PUMCH‐A‐210).”

We apologize for this error.

